# The Association between EGFR and cMET Expression and Phosphorylation and Its Prognostic Implication in Patients with Breast Cancer

**DOI:** 10.1371/journal.pone.0152585

**Published:** 2016-04-07

**Authors:** Young Kwang Chae, Debora de Melo Gagliato, Sachin Gopalkrishna Pai, Benedito Carneiro, Nisha Mohindra, Francis Joseph Giles, Praveen Ramakrishnan-Geethakumari, Joohyuk Sohn, Shuying Liu, Huiqin Chen, Naoto Ueno, Gabriel Hortobagyi, Ana Maria Gonzalez-Angulo

**Affiliations:** 1 Department of Breast Medical Oncology, The University of Texas M. D. Anderson Cancer Center, Houston, TX, United States of America; 2 Northwestern Medicine Developmental Therapeutics Institute, Northwestern University, Chicago, IL, United States of America; 3 Robert H. Lurie Comprehensive Cancer Center, Northwestern University, Chicago, IL, United States of America; 4 Division of Hematology and Oncology, Department of Medicine, Feinberg School of Medicine, Northwestern University, Chicago, IL, United States of America; 5 Department of Clinical Oncology, Hospital Sirio Libanes, São Paulo, SP, Brazil; 6 Instituto de Cancer do Estado de São Paulo (ICESP), São Paulo, SP, Brazil; University of California Davis, UNITED STATES

## Abstract

EGFR and cMET cross-talk is involved in breast cancer (BC) progression and resistance to different targeted therapies, however little is known about the co-expression patterns of EGFR and cMET or its prognostic significance in BC. Protein levels of EGFR, cMET and their phosphorylated proteins were measured in 825 BC samples using reverse phase protein array (RPPA). Given unimodal distribution of proteins, the median was selected as a cut-off after sensitivity analyses. Kaplan-Meier survival curves were used to estimate relapse-free (RFS) and overall survival (OS). Cox-proportional hazards models were utilized to determine associations between EGFR and cMET with outcomes. Mean age was 58 years with 457 (55%) hormone receptor (HR) positive, 211 (26%) triple-negative (TN) and 148 (18%) HER2 positive tumors (HER2+). HER2+ was associated with higher EGFR expression and phosphorylation, compared to HR and TN (p<0.05). High EGFR expression was associated with higher phosphorylated-cMET (p-cMET) but not cMET (ANOVA p-cMET p < 0.001; cMET p = 0.34). The same association was found with high phosphorylated-EGFR (p-EGFR) group at Tyr992 and Tyr1068 (both p < 0.001). High expressions in either of two p-EGFRs were linked with higher cMET as well (all p<0.001). For the TN subtype, high expression in EGFR and p-EGFR at Tyr992 but not at Tyr1068 was associated with higher p-cMET (p<0.00, p = 0.012, p = 0.4 respectively). Only high expression in p-EGFR at Tyr992 was linked with higher expression of cMET (p = 0.02). In contrast, among HER2 subtype, high expression in p-EGFR at Tyr1068 but not at Tyr992 was associated with higher cMET and p-cMET (cMET p = 0.023;p-cMET p<0.001). Four subgroups of patients defined by dichotomized EGFR/p-EGFR and cMET/p-cMET level demonstrated no significant differences in survival. In multivariate analyses, neither cMET nor EGFR expression/activation was found to be an independent prognostic factor in survival outcome.

## Introduction

Breast cancer is the most common invasive cancer and the second leading cause of cancer death in women worldwide[1]. Metastatic disease is still associated with a poor 5-year survival rate despite recent advances in therapy[2]. Improving our understanding of the molecular pathways in carcinogenesis and targeted therapeutics is extremely important in trying to overcome disease resistance mechanisms and to improve patients prognosis[1].

In the mid-1980’s, the proto-oncogene *MET* (on chromosome 7q31) was characterized and shortly thereafter the receptor tyrosine kinase (RTK) and its ligand, the hepatocyte growth factor/ scatter factor (HGF/SF), were described[3]. cMET, a transmembrane α/β heterodimer protein receptor, dimerizes and undergoes autophosphorylation upon binding of HGF. Consequently, proliferative intracellular downstream pathways are activated, such as the Ras-Erk/MAPK and the PI3K-Akt cascades, and promote invasive growth and apoptosis inhibition[4].

The role of HGF/SF and *MET* in cancer has been brought to light by several studies. The normally tightly regulated HGF/MET signaling axis is altered at multiple levels in tumorigenesis[5]. Transcriptional deregulation constitutes one the most important mechanisms to disease modulation. However, other alterations such as genomic amplification, activating point mutations, inadequate degradation and receptor crosstalk also may contribute[6]. Aberrations in the cMET pathway are also thought to play a role in the progression and invasive growth of several malignancies such as lung, kidney, head and neck, breast, and colorectal cancers[7–10].

Multiple studies have demonstrated a strong relationship between HGF/MET signaling and breast cancer progression. MET overexpression has been associated with an invasive phenotype during breast cancer progression *in vivo* and in animal models[8, 11]. Elevated tyrosine phosphorylation of cMET has been described in basal-like tumors[12]. We have previously reported that both cMET expression and phosphorylation were associated with worse outcomes, including inferior overall survival in all BC subtypes[13]. For instance, patients with high cMET had significantly higher risk of recurrence (HR: 2.06, P = 0.03) and death (HR: 2.81, P = 0.02). Our group also demonstrated that heavily pretreated metastatic BC patients who were found to display *MET* mutations or amplifications had frequent high-grade histology, higher metastatic disease burden, and inferior outcomes during phase I clinical trials[14].

“RTK coactivation” and “oncogene switching” are two important mechanisms that cancer cells utilize to evade normal cellular processing and clearance. Therefore, chemo-resistance and tumor progression can result from this coactivation[15]. Functional cross talk between *MET* and other signaling pathways, such as EGFR, ERBB2 and insulin like growth factor 1 receptor are examples from this interaction. EGFR, cMET and their cross talk play an important role in cancer progression and development of resistance to different targeted therapies[16, 17]. In fact, acquired resistance to gefitinib, an EGFR tyrosine kinase inhibitor, can be mediated by *MET* amplification in patients with non-small cell lung cancer (NSCLC)[18, 19]. In HER2-overexpressing breast cancer cells, MET aberrations also contribute to trastuzumab resistance [20]. In addition, basal BC cell lines demonstrated differential responsiveness to small molecule inhibitors of cMET and EGFR and the magnitude of response correlated with the degree of target phosphorylation[12]. Based on these results, EGFR and cMET dual blockade has been proposed as an attractive targeted therapy for BC[15]. This approach suggests that inhibiting co-activated network kinases may enhance efficacy and overcome resistance mechanisms.

However, little is known about the co-expression patterns of EGFR and cMET in human BC and its prognostic significance. We hypothesized that EGFR and cMET might be co-expressed or co-activated in BC resulting in adverse survival outcomes. Using functional proteomic profiling we investigated the association between the expression pattern of both EGFR/cMET and p-EGFR/cMET proteins and survival outcome in all subtypes of BC.

## Methods

### Patient Material

Fine needle aspirates or mastectomy samples from 825 primary invasive BC were obtained. The samples were previously collected from tumor specimens in patients treated at the University of Texas MD Anderson Cancer Center between 1986 and 2007. Institutional Review Board (IRB) of the University of Texas MD Anderson Cancer Center approved the protocol and specimen obtained from repository. The corresponding clinical data was obtained from the Breast Cancer Management System database at our institution. Electronic chart review supplemented deficient information from the medical database. Patient records/information including all specimens were anonymized and de-identified prior to analysis.

Samples were categorized into three clinically relevant BC subtypes by immunohistochemistry (IHC) for estrogen receptor (ER) and progesterone receptor (PR) status and by IHC or fluorescent in situ hybridization (FISH) for HER2 status as per American Society of Clinical Oncology and College of American Pathologists (ASCO/CAP) guidelines[21]. Hormone receptor-positive (HR+) tumors were ER-positive and/or PR-positive and HER2-negative. Correspondingly, HER2-positive (HER2+) group included all HER2 positive tumors, irrespective of hormone receptor status. Triple negative (TN) subgroup comprised all cases with ER/PR and HER2 status negative.

### Reverse Phase Protein Microarray (RPPA)

RPPA was performed from proteins extracted from human tumors in our laboratory, as described previously[22]. Lysis buffer was used to lyse frozen tumors via homogenization. Tumor lysates were normalized to 1 μg/μL concentration using bicinchoninic acid assay and boiled with 1% SDS, and the supernatants were manually diluted in six or eight 2-fold serial dilutions with lysis buffer. An Aushon Biosystems (Burlington, MA) 2470 arrayer created 1,056 sample arrays on nitrocellulose-coated FAST slides (Schleicher & Schuell BioScience, Inc.) from the serial dilutions. A slide was then probed with validated primary cMET and p-cMET antibodies (Cell Signaling Technology, Danvers, MA). The signal was amplified using a DakoCytomation–catalyzed system. The antibodies for cMET (Mouse) and p-cMET (Rabbit, Y1235) were used at a dilution of 1:250 for RPPA. A secondary antibody was used as a starting point for amplification. The representative RPPA slide is shown in the supporting information (S1 Fig).

The slides were scanned, analyzed, and quantitated using Microvigene software (VigeneTech Inc.) to generate serial dilution–signal intensity curves for each sample. A representative natural logarithmic value of each sample curve on the slide (curve average) was then used as a relative quantification of the amount of each protein in a given sample. The level of cMET and p-cMET in each sample was expressed as a log mean centered value, after correction for protein loading, using the average expression levels of over 150 proteins as previously described[22].

### Statistical Methods

Boxplots were created for original and log2 transformed expressions of total cMET and p-cMET according to BC subtypes. The original expressions were right-skewed. However, the log2 transformation data was normally distributed. Hence, all following statistical analyses were generated with the log2 transformation of the original expression values. All tests were two-sided. P values less than 0.05 were considered statistically significant. Statistical analyses were done with R statistical software version 2.12.0 (R Development Core Team, Vienna, Austria).

Mean and standard deviations were generated for total cMET and p-cMET by tumor subtype. Given unimodal distribution of proteins, median was selected as a cut-off after sensitivity analyses. Overall survival (OS) was measured from the date of diagnosis to the date of death or lost to follow-up. Relapse-free survival (RFS) was defined from the time of diagnosis to first relapse. Median RFS and OS were estimated nonparametrically with the use of Kaplan-Meier curves by levels of total cMET/EGFR and p-cMET/EGFR expression and compared by the log-rank statistic. Cox proportional hazards models were fit to determine associations of EGFR and cMET with outcomes after adjustment for other clinical characteristics, such as age, tumor stage, nodal status, receptor subtype and histologic grade.

## Results

### EGFR and cMET Expression and Phosphorylation Patterns

Mean age of the patient population was 58 years. There were 457 (55%) HR+, 211 (26%) triple-negative (TN) and 148 (18%) HER2+ tumors. The HER2+ subgroup was found to display higher EGFR expression and phosphorylation, compared to HR+ and TN subtypes (p<0.05).

Table 1 demonstrates the association between the expression levels of EGFR/p-EGFR and the cMET/p-cMET (ANOVA test used by the dichotomized expression groups in high and low expression levels). High EGFR expression was associated with higher p-cMET, but not cMET, compared to the low expression group (ANOVA p-cMET p<0.001, MET p = 0.34). EGFR expression also correlated with p-cMET in HR + and TN subgroups, but not in the HER2+ subgroup. The expression of both p-EGFR (Tyr992 and Thy1069) correlated with the expression of p-cMET (p<0.01) in the overall sample and the HR+ subtype.

**Table 1:** Patterns of correlation between EGFR and cMET expression and phosphorylation in all breast cancers and subtypes.

	All		HR		TN		HER2	
	cMET	p-cMET	cMET	p-cMET	cMET	p-cMET	cMET	p-cMET
EGFR	-	+	-	+	-	+	-	-
p-EGFR Tyr992	-	+	+	+	+	+	-	-
p-EGFR Tyr1068	-	+	+	+	-	-	+	+

+ = statistically significant positive association; - = no statistically significant association

However, the correlation between p-EGFR and cMET/p-cMET was distinct between TN and HER2 subgroups. The expression of p-EGFR Tyr992 was significantly associated with both cMET and p-cMET among TN cases (p<0.05), but not among HER2 positive breast cancer. On the other hand, p-EGFR Tyr1068 expression correlated only with HER2 positive breast cancer cases (p<0.05) and not with TN. These results suggest possible association between co-activation patterns of EGFR and cMET and the subgroups of breast cancer evaluated.

**Fig 1:**
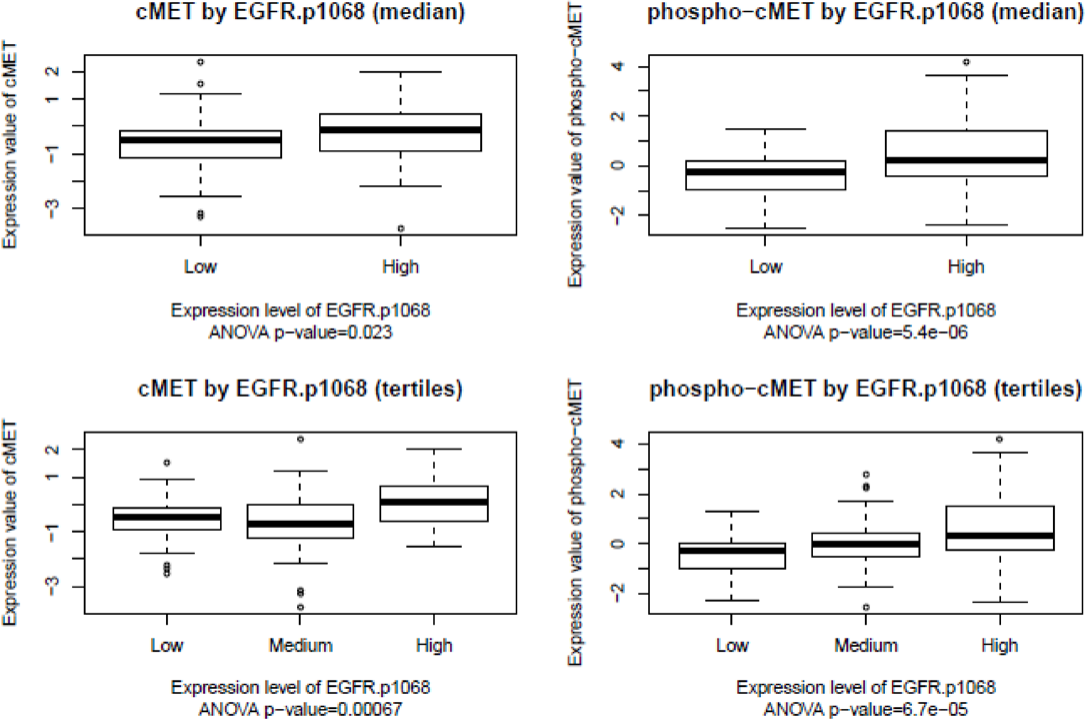
Boxplot of cMET and p-cMET by EGFR phosphorylation. The boxplots of expression and phosphorylation values of cMET with different expression levels of EGFR.p1068 in HER2 positive breast cancers is shown. The cutoffs of EGFR.p1068 expression levels were based on median or tertiles of the expression values of EGFR.p1068. The ANOVA p-values testing comparison significance are shown.

**Fig 2:**
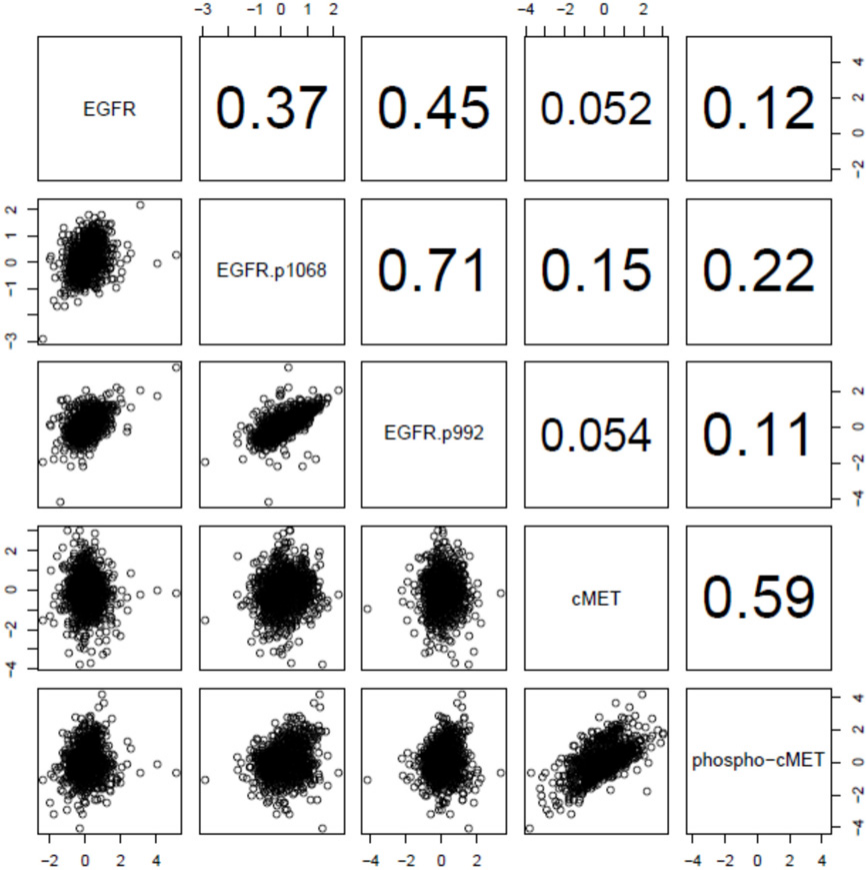
Pairwise scatter plot of EGFR and cMET and their phosphorylated forms in all breast cancers. The upper triangle shows the Spearman correlation coefficients between pairs of proteins.

Fig 1 & Fig 2 exhibit the relationship of EGFR and cMET expression in a different visual fashion, using the dot plots and Spearman’s correlation coefficients as an indicator for the strength in association respectively. Strong associations were observed, as expected, between EGFR and p-EGFR, and between cMET and p-cMET. The strongest association was found between the two p-EGFRs (Spearman’s coefficient = 0.71). Interestingly, the association between the total expression and phosphorylation was stronger in cMET, when compared to EGFR. The correlation coefficients between EGFR/p-EGFR and cMET/p-cMET are low in numbers, reflecting weak associations between the two receptors.

### Survival Outcomes

**Fig 3:**
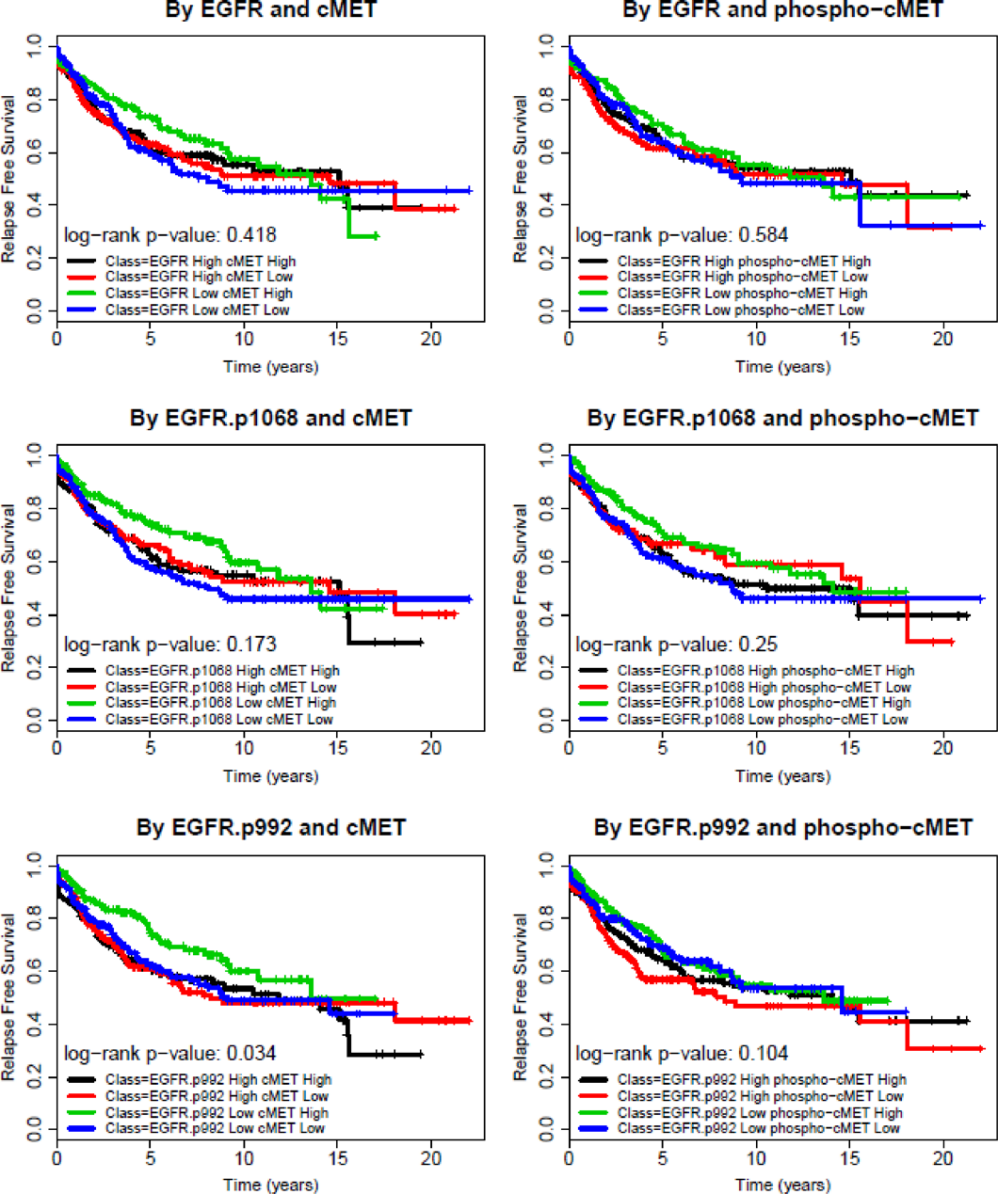
Relapse-free survival of breast cancer patients by EGFR and cMET/p-cMET status. Kaplan-Meier RFS in all breast cancers; Samples were grouped by expression levels of EGFR and cMET or their phosphorylated forms (Low: below median High: above median).

**Fig 4:**
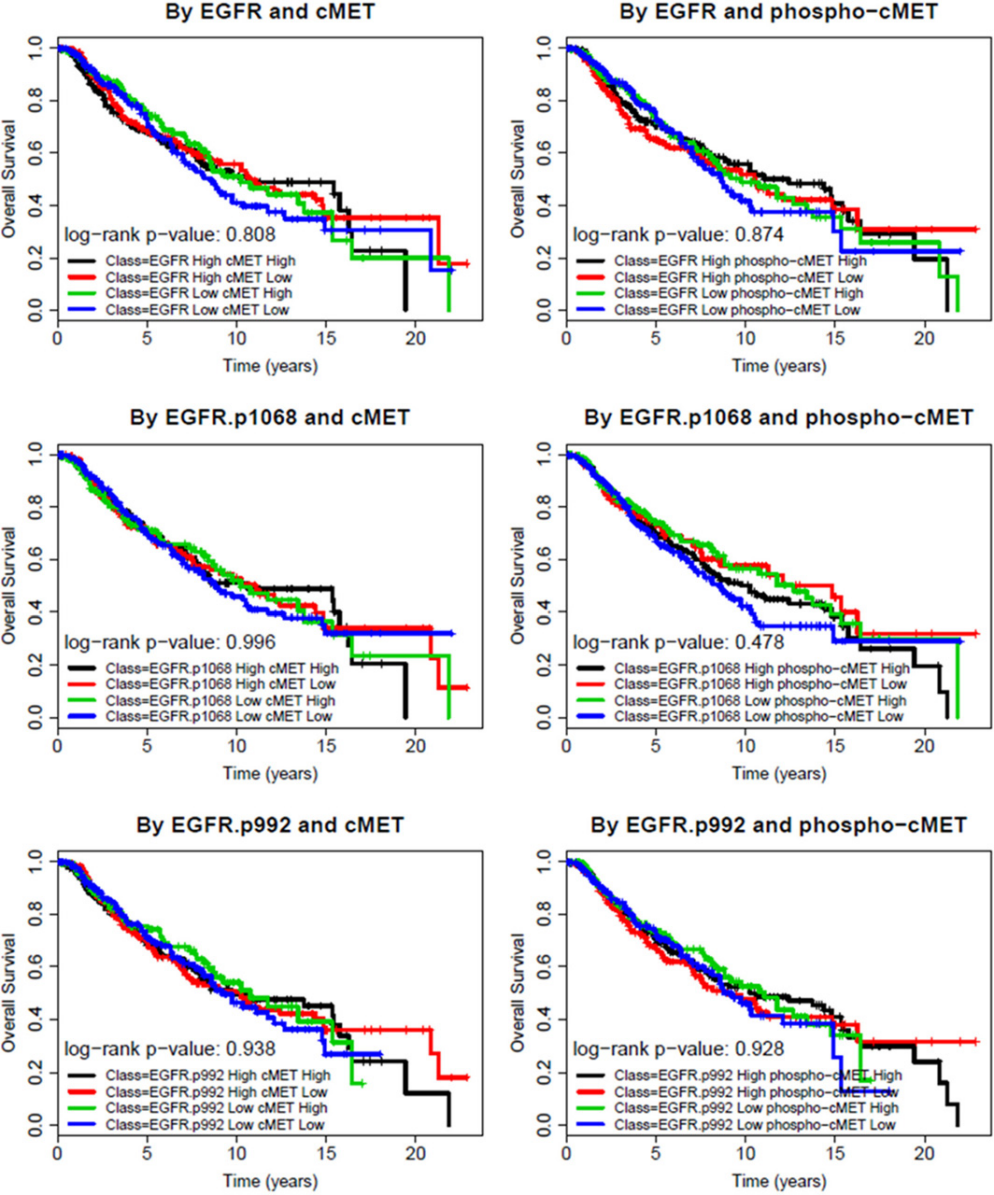
Overall survival of breast cancer patients by EGFR and cMET/p-cMET status. Kaplan-Meier OS in all breast cancers; Samples were grouped by expression levels of EGFR and cMET or their phosphorylated forms (Low: below median High: above median).

Patients were divided into four different groups by dichotomized EGFR and cMET expression status (high, >50% and low, <50%): ‘high-high’, ‘high-low’, and ‘low-high’, and ‘low-low’ groups. The same approach was used for two p-EGFR and p-cMET levels resulting in six different paired combinations. Kaplan-Meir survival analyses revealed that none of the combinations within the four comparison groups had a statistically significant difference in survival outcomes. Fig 3 demonstrates no meaningful difference in RFS among the four groups by EGFR and cMET expression or phosphorylation status, except for p-EGFR at Tyr1068. Patients with tumor samples that had low p-EGFR at Tyr992 and high cMET experienced favorable RFS (log rank test p = 0.034). Fig 4 also illustrates no significant difference in OS among the different groups mentioned above. The same method was used to compare RFS and OS in subgroups with different levels of expression of EGFR/p-EGFR and cMET/p-cMET among HR+, HER2+ and TN BCs. Again, no difference in survival was found in these subgroup analyses.

Multivariate Cox proportional hazard model was used to assess the risk of relapse and overall survival in patients with different EGFR/cMET expression and activation patterns after controlling for demographic and clinico-pathological variables including age, tumor stage, nodal status, receptor subtype and histologic grade. The levels of p-cMET, EGFR, and two p-EGFRs did not show significant association with any survival outcomes. After multivariate analyses, neither cMET nor EGFR expression or activation was found to be an independent prognostic factor in survival outcome.

## Discussion

We found that EGFR and cMET are frequently co-expressed and/or co-activated in human BC and this pattern varied according to BC subtypes. Furthermore, EGFR (or p-EGFR) was more likely to correlate significantly with p-cMET than with un-phosphorylated cMET.

The HER2+ subtype was found to display higher EGFR expression and activation compared to other subtypes. The TN subgroup had high expression of EGFR and p-EGFR at Tyr992.

In our cohort, co-expression or co-phosphorylation of cMET and EGFR were not associated with adverse survival outcomes. However in TNBC tumors, co-expression of MET and EGFR has been linked to significantly worse DFS and a trend towards worse OS compared to expression of EGFR alone[23]. In our study, cMET was found to be an independent prognostic factor, irrespective of EGFR expression or phosphorylation status, with higher cMET expression associated with adverse outcome. A meta-analysis of 21 published studies between 1998 and 2014, comprising a total of 6010 cases, showed that c-Met overexpression is a statistically significant adverse predictor of RFS and OS in unselected breast cancer[24].

A relationship between higher EGFR expression and decreased survival in BC patients has been described as well as an association between EGFR expression and undifferentiated tumor cells[25]. The evaluation of EGFR and HER2 expression by IHC among 105 BC samples showed that EGFR staining was associated with elevated rates of cell proliferation (measured by percentage of Ki67 positive cells). EGFR expression was associated with HER2 expression by immunohistochemistry and worsened patient outcome, as well as hormone insensitivity in moderately EGFR positive samples[26]. Furthermore, EGFR expression was associated with loss of endocrine sensitivity in BC[27, 28]. These results corroborate the notion that EGFR mediates important pathways related to tumor aggressiveness and lack of response to therapy. Consequently, breast cancer tumors overexpressing EGFR might be associated with unfavorable survival outcomes.

Our results suggest possible distinct interactions and co-activation patterns of EGFR and cMET among the subgroups of breast cancer. Similar findings between these pathways have also been described in other tumor types. In a glioblastoma xenograft cell model [29], elevated levels of mRNA HGF and cMET were detected in the mesenchymal subtype of glioblastoma, a more aggressive form of this disease. Activation of cMET and HGF expression was found in tumors with an activating mutation in EGFR. Also, in anaplastic thyroid carcinoma cell lines, constitutive activation of cMET was found simultaneously with elevated expression of EGFR. Additionally, an EGFR tyrosine kinase inhibitor was capable of de-activating EGFR pathway and down-regulating cMET expression[30]. This crosstalk between EGFR and cMET was one of the mechanisms proposed to explain the constitutive activation of cMET in the absence of its ligand, HGF[31]. It is suggested that TGFα pathway and the autocrine activation of EGFR, leading to tumor growth, apoptosis inhibition and metastasis play a role in this process.

In our study, after multivariate analyses controlling for various clinicopathological variables, cMET expression was not found to be an independent prognostic factor in survival outcome. This contrasts with previous research findings in BC, which clearly demonstrated that cMET alterations were associated with unfavorable survival outcomes in BC. It is unclear whether this is due to random effect or limited sample size.

Poorly differentiated and invasive BC cell lines with increased motility and invasiveness were associated with high levels of cMET receptor[32]. Thus, our group evaluated levels of cMET and p-cMET in 257 samples by RPPA and demonstrated that both cMET and p-cMET were independent prognostic factors for RFS (HR 2.44, 95% confidence interval (CI): 1.34–4.44, P = 0.003) and also for OS (HR: 3.18, 95% CI: 1.43–7.11, P = 0.003).[13] Furthermore, in a heavily pretreated metastatic BC patient cohort (those referred to our phase I oncology department), tumors harboring *MET* aberrations were found to have unfavorable survival outcomes, compared to those without *MET* alterations[14], corroborating our findings that cMET alteration is a poor prognostic factor.

Our results demonstrated that the TN subtype had high expression of EGFR and p-EGFR at Tyr992, which was associated with higher expression of cMET. The significance of EGFR expression in TNBC and its prognostic relevance are of particular interest in this subgroup of patients.

EGFR protein expression, gene copy number alteration and mutation in the exons 18 to 21 were examined in 151 cases of TNBC patients and these findings were correlated with clinical outcomes[33]. High EGFR copy number, but not EGFR mutation, correlated with EGFR protein overexpression, which was found in 95 (64%) of the cases. Patients whose tumors had EGFR amplification experienced poor disease-free survival, suggesting that this marker might be useful for predicting outcomes in these patients. Breast tumors expressing EGFR presented higher proliferation rates and were more likely to be grade III and estrogen receptor negative[34]. These results suggested a role for EGFR in the pathogenesis of TNBC and provided rationale for the clinical investigation of anti-EGFR monoclonal antibodies (i.e. cetuximab) in the treatment of TNBC. A phase II trial randomized 115 patients to receive cetuximab plus cisplatin or cisplatin alone[35]. Overall response rate was 20% for combination therapy compared with 10% for cisplatin monotherapy (OR, 2.13; 95% CI, 0.81 to 5.59; P = .11). Cisplatin plus cetuximab also resulted in longer PFS compared with cisplatin alone (median, 3.7 v 1.5 months; HR 0.67; 95% CI, 0.47 to 0.97; P = .032).

However, other trials that combined cetuximab with other chemotherapeutic agents, such as carboplatin[36] and ixabepilone[37], failed to demonstrate a significant benefit with combination therapy.

Overall, our results demonstrate that EGFR and cMET are either frequently co-expressed or co-activated in human BC. Multivariate analyses demonstrated that neither cMET nor EGFR expression or activation was found to be an independent prognostic factor in survival outcome. Future clinical trials designed to recruit this particular group of patients are needed.

## Supporting Information

S1 Fig Reverse phase protein assay (RPPA) slide. A representative figure of reverse phase protein assay (RPPA) slide is shown.
(TIF)
