# Supplementary figures and images for: The Association between EGFR and cMET Expression and Phosphorylation and Its Prognostic Implication in Patients with Breast Cancer
Source: PLoS One. 2016 Apr 7;11(4):e0152585. doi: 10.1371/journal.pone.0152585 (PMC4824503; doi:10.1371/journal.pone.0152585)

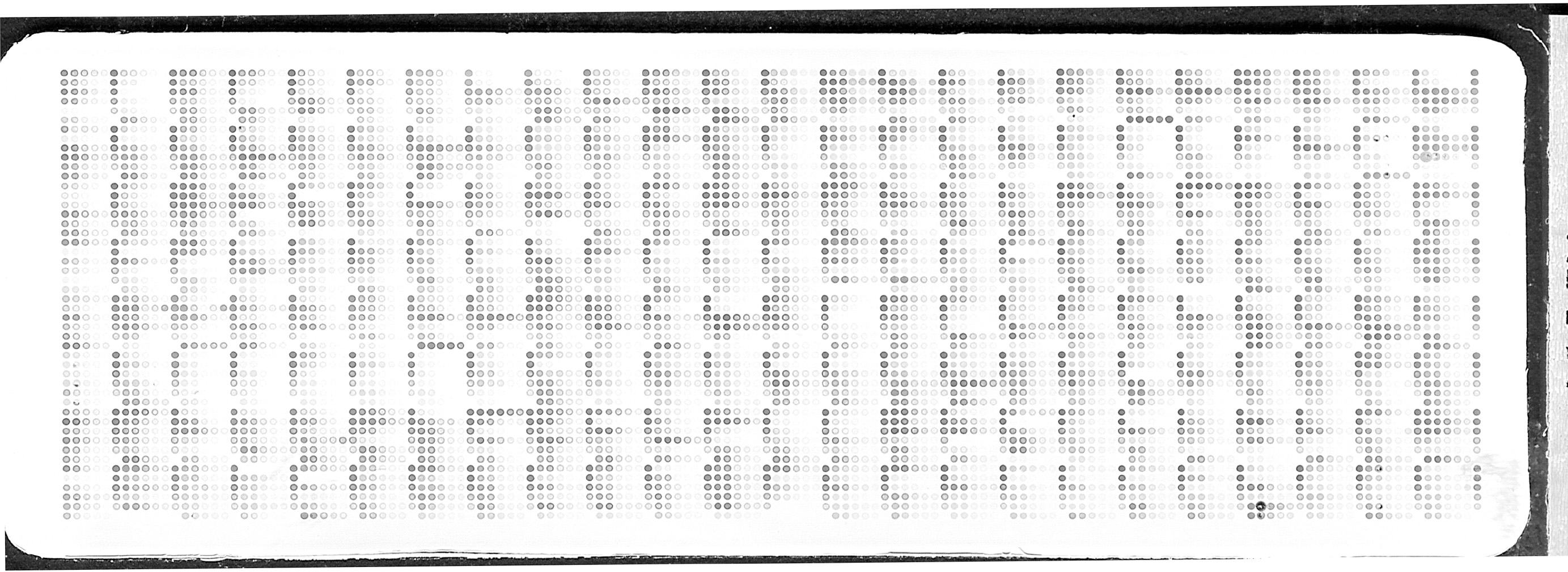

Supplement: S1 Fig — A representative figure of reverse phase protein assay (RPPA) slide is shown. (TIF) [file pone.0152585.s001.tif]
